# Supplementary material for: A Critical View of the Application of the APEX Software (Aqueous Photochemistry of Environmentally-Occurring Xenobiotics) to Predict Photoreaction Kinetics in Surface Freshwaters
Source: Molecules. 2019 Dec 18;25(1):9. doi: 10.3390/molecules25010009 (PMC7017383; doi:10.3390/molecules25010009)
Supplement: Supplementary file 1 [file molecules-25-00009-s001.zip › 3.2.4_gcc-4.4.0.zip!/3.2.4_gcc-4.4.0/3.2.4_gcc-4.4.0/doc/octave/html/interpreter/Object-Oriented-Programming.html]

Object Oriented Programming - Untitled


Next: System Utilities,
Previous: Audio Processing,
Up: Top


---

## 33 Object Oriented Programming

Octave includes the capability to include user classes, including the
features of operator and function overloading. Equally a user class
can be used to encapsulate certain properties of the class so that
they cannot be altered accidentally and can be set up to address the
issue of class precedence in mixed class operations.

This chapter discussions the means of constructing a user class with
the example of a polynomial class, how to query and set the properties
of this class, together with the means to overload operators and
functions.

- Creating a Class- Manipulating Classes- Indexing Objects- Overloading Objects- Inheritance and Aggregation
